# Antioxidant Activity of Selected Polyphenolics in Yeast Cells: The Case Study of Montenegrin Merlot Wine

**DOI:** 10.3390/molecules23081971

**Published:** 2018-08-07

**Authors:** Neda O. Đorđević, Nevena Todorović, Irena T. Novaković, Lato L. Pezo, Boris Pejin, Vesna Maraš, Vele V. Tešević, Snežana B. Pajović

**Affiliations:** 1 Laboratory of Molecular Biology and Endocrinology, Institute of Nuclear Sciences Vinča, University of Belgrade, 11001 Belgrade, Serbia; nevenat@vin.bg.ac.rs (N.T.); pajovic@vin.bg.ac.rs (S.B.P.); 2 Centre of Chemistry, Institute of Chemistry, Technology and Metallurgy, University of Belgrade, 11000 Belgrade, Serbia; irenan2003@yahoo.com; 3 Institute of General and Physical Chemistry, University of Belgrade, 11000 Belgrade, Serbia; latopezo@yahoo.co.uk; 4 Department of Life Science, Institute for Multidisciplinary Research—IMSI, University of Belgrade, 11000 Belgrade, Serbia; brspjn@gmail.com; 5 Sector for Development, 13. Jul Plantaže, 81000 Podgorica, Montenegro; vesna.maras@plantaze.com; 6 Faculty of Chemistry, University of Belgrade, 11000 Belgrade, Serbia; vtesevic@chem.bg.ac.rs; 7 Faculty of Medicine, University of Niš, 18000 Niš, Serbia

**Keywords:** red wine, polyphenolics, antioxidant activity, *Saccharomyces cerevisiae*, artificial neural network

## Abstract

Screens of antioxidant activity (AA) of various natural products have been a focus of the research community worldwide. This work aimed to differentiate selected samples of Merlot wines originated from Montenegro, with regard to phenolic profile and antioxidant capacity studied by survival rate, total sulfhydryl groups and activities of glutathione peroxidase (GPx), glutathione reductase and catalase in H_2_O_2_–stressed *Saccharomyces cerevisiae* cells. In this study, DPPH assay was also performed. Higher total phenolic content leads to an enhanced AA under both conditions. The same trend was observed for catechin and gallic acid, the most abundant phenolics in the examined wine samples. Finally, the findings of an Artificial Neural Network (ANN) model were in a good agreement (*r*^2^ = 0.978) with the experimental data. All tested samples exhibited a protective effect in H_2_O_2_–stressed yeast cells. Pre-treatment with examined wines increased survival in H_2_O_2_–stressed cells and shifted antioxidative defense towards GPx–mediated defense. Finally, sensitivity analysis of obtained ANN model highlights the complexity of the impact that variations in the concentrations of specific phenolic components have on the antioxidant defense system.

## 1. Introduction

The beneficial effect of moderate red wine consumption on human health has been recognized for a long time. At least in part, this protective effect is mediated by polyphenolic antioxidants [1,2]. These compounds display a large array of bioactivities including free radical–scavenging and/or neutralizing, metal chelating, cell signaling pathway modulation, gene expression along with antioxidant enzyme modulation [3,4,5,6]. One of the mechanisms of their antioxidant activity (AA) is based on the activation of the antioxidant enzymes such as glutathione peroxidase (GPx), glutathione reductase (GR) and catalase (Cat) [7].

Due to the complexity of biological systems, chemical AA assays are increasingly being replaced with the cellular ones that consider the issues of bioavailability and metabolism. Indeed, the eukaryotic yeast *Saccharomyces cerevisiae* represents a good model system that has been previously used for a rapid determination of AA of food [7,8].

In order to fight against oxidative stress, yeast cells are equipped with a number of antioxidant enzymes, including GPx, GR and Cat. Glutathione (GSH), the most abundant thiol in cells, represents the first line of defense against oxidative stress. Furthermore, GPx catalyses the reduction of H_2_O_2_ and a wide variety of organic peroxides to water and the corresponding stable alcohols using GSH as a source of electron [9]. This results in GSH oxidation to glutathione disulfide (GSSG) which is reduced back to GSH by GR. Thus, this enzyme is responsible both for recycling of GSH (consumed by GPx) and maintenance of a high reduced/oxidized ratio inside the cell [10]. Like GPx, Cat also protects cells from the toxic effect of H_2_O_2_.

In recent years, the results of the mathematical modeling have been increasingly used for the study of AA [11,12], and the developed models showed a good fit to experimental data. Nonlinear models are found to be more suitable for real process simulation. Artificial neural network (ANN) models are perceived as a good modeling tool since they provide an empirical solution to the problems from a set of experimental data. In addition to this, they are capable of handling complex systems with non–linearities and interactions between decision variables [12,13,14,15].

This work aimed to differentiate selected samples of Merlot wines originated from Montenegro, with regard to phenolic profile and antioxidant capacity studied by anti-DPPH radical activity, survival rate (SR), total sulfhydryl groups (T–SH) content and activities of GPx, GR and Cat in H_2_O_2_–stressed *Saccharomyces cerevisiae* cells. Besides, we aimed to characterize and differentiate the examined wine samples, as well as to develop an ANN model for AA prediction, based on phenolic content in wine. For that purpose, the commercial Merlot wine, along with red wine samples obtained from recognized clones (VCR 1 and VCR 101) of the same variety (vintage 2011) were used. The samples were labeled as Comm, C I and C II, for the samples of the commercial wine, VCR 1 and VCR 101 clone wines, respectively.

## 2. Results and Discussion

### 2.1. Phenolic Profile and In Vitro Antioxidant Activity

According to Total Phenolic Content (TPC), there was no significant differences between clone I (C I) and clone II (C II) wines. However, compared to commercial wine (Comm), C II wine was enriched with TPC (*p* < 0.05) (Table 1). While similar TFC was found in all analyzed samples (*p* > 0.05), the sample C II was enriched with Total Monomeric Anthocyanin Content (TMA) (*p* < 0.05) (Table 1).

Catechin (C) and gallic Acid (GA) were the most abundant phenolics in the examined wine samples [16]. Their highest/lowest concentration were noticed for the samples C I and Comm, respectively. In addition to this, epicatechin (EC) values of the wine samples C I and C II were significantly higher than the commercial one. Finally, the similar trend was observed for quercetin (Qe), myricetin (My) as well as *trans*- and *cis*-resveratrol (tR and cR) and *trans*- and *cis*-piceid (tP and cP) [16]. Interestingly, Comm wine contained more than ten times higher content of caffeic acid (CA) compared to C I and C II samples. Since the beneficial effects of these compounds on human health has been confirmed by previous studies [17,18,19,20,21,22,23,24], given wines receive additional nutritive value.

**Figure 1:**
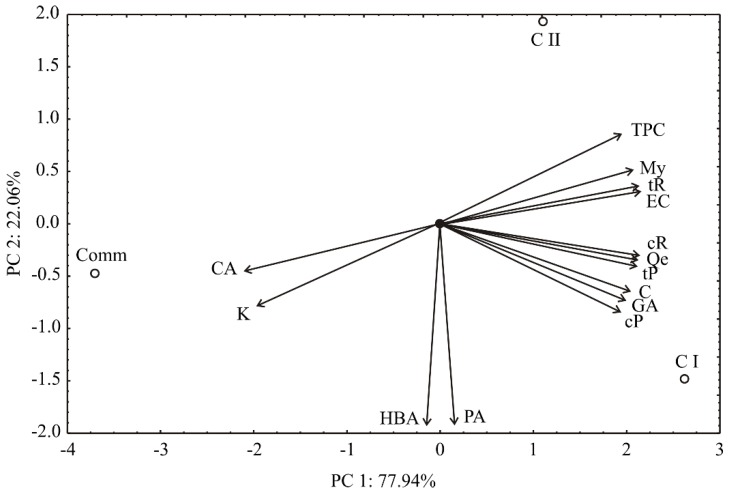
Biplot graph of phenol content found in wines. Comm—commercial wine; C I—clone I wine; C II—clone II wine; TPC—Total phenolic content; GA—Gallic acid; PA—Protocatechuic acid; HBA—4-Hydroxybenzoic acid; CA—Caffeic acid; C—Catechin; EC—Epicatechin; tR—*trans*-Resveratrol; cR—*cis*-Resveratrol; tP—*trans*-Piceid; cP—*cis*-Piceid; My—Myricetin, Qe—Quercetin; K—Kaempferol.

According to Principal Component Analysis (PCA), the first two components accounted for 100% of the total variance (77.94 and 22.06%, respectively) in the fourteen variables (analyzed polyphenolics) (Figure 1). According to a first principal component, the contents of TPC (which contributed 7.4% of total variance, based on correlations), My (8.5%), tR (8.8%), EC (8.9%), cR (8.8%), Qe (8.9%), tP (8.8%), C (8.1%), GA (7.8%) and cP (7.4%) exhibited positive scores. On the other hand, negative score values were observed for CA (8.7%) and kaempferol (K) (7.7%), according to first principal component. According to the second principal component, negative and positive contributions were observed for protocatechuic acid (PA) (32.2% of total variance, based on correlations) and 4-hydroxybenzoic acid (HBA) (32.2%) and TPC (7.3%), respectively.

The points presented in the PCA graph indicate the similarity of the samples. The positive correlation between C and GA (the most abundant phenolic compounds in the analyzed samples) and GA and cP respectively were observed (*p* < 0.05). The content of CA is negatively correlated to the content of EC, tR and My (*p* < 0.05), while EC is positively correlated to tR (*p* < 0.05). Finally, cR is positively correlated to tP and Qe (*p* < 0.05). PCA graphic quite well made discrimination between the samples (Figure 1). Those with higher TPC, My, tR, EC, cR, Qe, tP, C, GA and cP content are located at the right side of the graph (C I and C II samples), while the sample Comm (enriched with CA and K) is located at the left side of the graph. The first principal component is described by TPC, My, tR, EC, cR, Qe, tP, C, GA, cP, CA and K content (the differentiation between samples is predominantly determined by these variables), while the second principal component is determined by the contents of HBA and PA.

**Figure 2:**
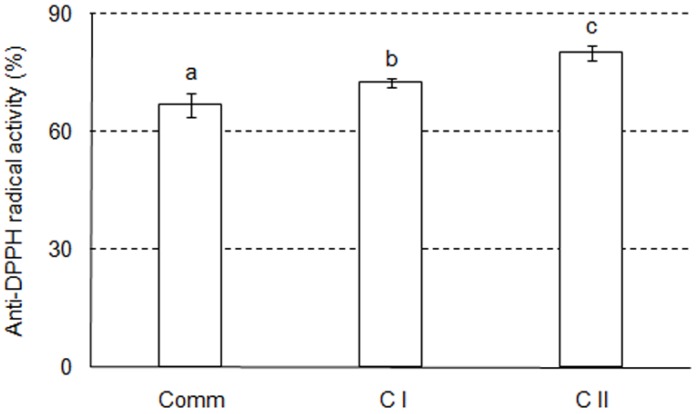
Anti–DPPH radical activity of analyzed samples. Results are expressed as IC_50_^−1^ values (mean ± SD), representing the reciprocal volume of the wine sample able to scavenge 50% of DPPH radical. Different letters show significant differences (*p* < 0.05) between obtained values, according to Tukey’s HSD test. Comm—commercial wine; C I—clone I wine; C II—clone II wine.

With regard to in vitro antioxidant capacity, Comm wine had the lowest anti–2,2-diphenyl-1-picrylhydrazyl (anti–DPPH) radical activity (Figure 2) which is in line with the TPC, as well as with the content of most phenolic compounds found in these samples. Anti–DPPH radical activity assay revealed that C II sample, which contained the highest level of the TMA, had the highest in vitro protective action. Positive correlation between anthocyanins and anti–DPPH radical activity was reported previously [25,26]. Anthocyanins, phenolic compounds widely distributed in berry fruits, act as antioxidants or free radical scavengers and have a wide range of health–promoting properties [27].

### 2.2. Antioxidant Activity in Cell Culture

**Figure 3:**
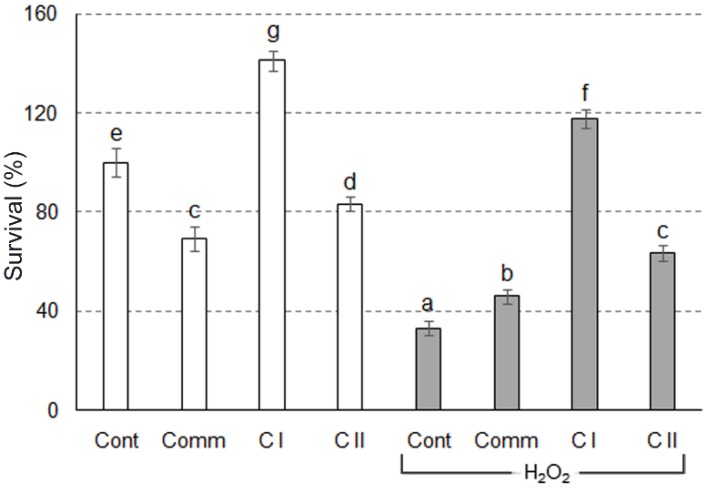
Survival rate of yeast cells. The results are expressed as mean ± SD. Different letters show significant differences between obtained values (*p* < 0.05), according to Tukey’s HSD test. Cont—negative control, cells treated with 13% ethanol; Cont + H_2_O_2_—positive control, cells treated with H_2_O_2_; Comm—commercial wine; C I—clone I wine; C II—clone II wine.

To examine protective effects of the examined wines, SR of H_2_O_2_–stressed yeast cells was tested (Figure 3). Compared to 13% ethanol (100%), H_2_O_2_ decreased survival rate of yeast cells to 33%. Though pretreatment with all wine samples increased survival in H_2_O_2_–stressed cells, C I sample exhibited the most prominent effect. However, C II sample stood out in performed DPPH assay analysis. Inconsistencies between results obtained in chemical and cellular AA assays have been reported to date [28,29]. In brief, there are still unresolved issues related to bioapsorption, bioavailability and metabolism of phenolics in cell culture [29]. Additionally, previous studies claimed there is a synergistic interaction between different phenolics, so the final AA may be different, compared to a contribution of each single compound [30].

**Figure 4:**
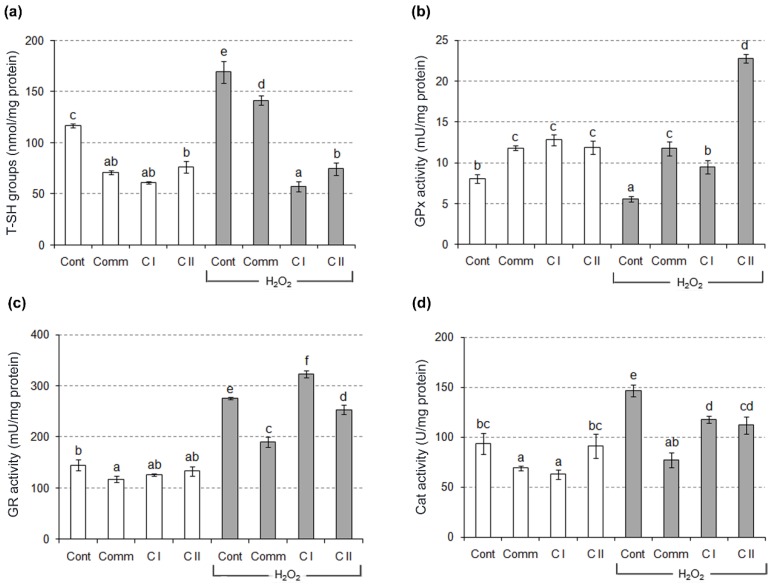
The content of total SH groups and enzyme activities in yeast cells. The results are expressed as mean ± SD. Different letters show significant differences between obtained values (*p* < 0.05), according to Tukey’s HSD test. T–SH—total sulfhydryl groups; GPx—glutathione peroxidase; GR—glutathione reductase; Cat—catalase; Cont—negative control, cells treated with 13% ethanol; Cont + H_2_O_2_—positive control, cells treated with H_2_O_2_; Comm—commercial wine; C I—clone I wine; C II—clone II wine.

Protective effects of the wines in the H_2_O_2_–stressed yeast cells were monitored through GPx, GR and Cat activities (Figure 4). A significant increase in Cat and the decrease in GPx activity was observed in H_2_O_2_–stressed cells. Though both enzymes catalyze reactions of H_2_O_2_ reduction, Cat predominantly took part in a defense response against oxidative attack. However, antioxidative defense was shifted towards GPx–mediated defense in all wine–treated yeast cells. In other words, pre-treatment with examined wine samples prevented H_2_O_2_–induced decrease of GPx activity, with C II sample as the most effective one. Compared to the control cells, GPx activity of pre–treated yeast cells was more than two–fold higher. Additionally, GR activity was significantly increased in H_2_O_2_–stressed cells, which was in line with increased level of T–SH groups. So, a decreased GPx activity may be primarily linked with some post-translational modification, rather than with lack of the cosubstrate GSH. Most likely, an increase of GPx activity actually resulted in a higher SR under oxidative stress. Such a finding is in a good agreement with previous ones [31,32].

**Figure 5:**
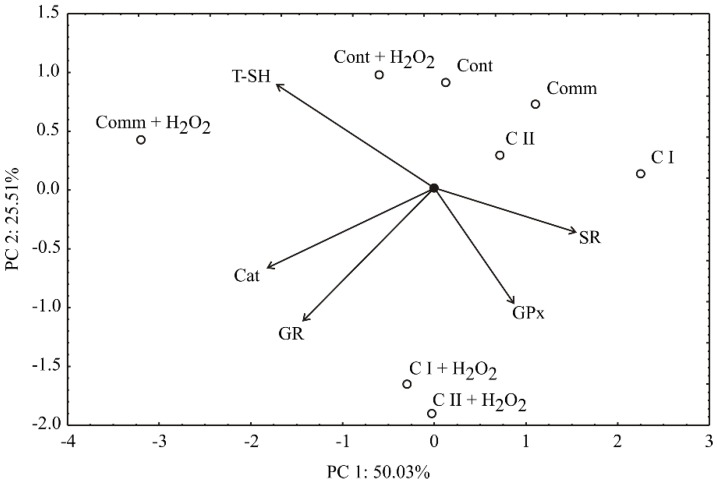
PCA ordination of variables based on component correlations, for antioxidant activity in wines. Comm—commercial wine; C I—clone I wine; C II—clone II wine; SR—survival rate; T–SH—total sulfhydryl groups; GPx—glutathione peroxidase; GR—glutathione reductase; Cat—catalase.

PCA of AA data pointed out that the first component accounted for 50.02% of the total variance, while the second component explained 25.51%, in the five variables (AA of wine samples) (Figure 5). Considering the PCA map, SR (which contributed 20.7% of the total variance, based on correlations) demonstrated a positive score (according to the first principal component), unlike GR (18.0%), Cat (29.4%) and T–SH (25.7%) which demonstrated a negative score value. Furthermore, a negative contribution of the second principal component was observed for GPx (26.0% of total variance, based on correlations), GR (35.9%) and Cat (13.1%), with T–SH as the only exception (21.2%, a positive score).

The PCA graph has enabled quite a good discrimination between the samples (Figure 5). The samples with higher T–SH, Cat and GR and GR and GPx are located at the left side and bottom of the graph, respectively (Comm + H_2_O_2_ and Cont + H_2_O_2_ and C I + H_2_O_2_ and C II + H_2_O_2_, respectively). The increased SR value is observed on the right side of the graph, where the samples Comm, C I and C II are located. Additionally, the smallest angle between SR and GPx indicates that GPx activity, in such a case, has the greatest positive effect on SR. In summary, all examined wine samples exhibited a protective effect in the yeast cells treated with H_2_O_2_.

### 2.3. Neurons in the ANN Hidden Layer

All variables considered for PCA were also used for ANN modeling. The optimum number of hidden neurons was chosen upon minimizing the difference between predicted ANN values and desired outputs, using sum of squares (SOS) during testing, as a performance indicator. According to the results, the optimal number of neurons in the hidden layer for SR, T–SH, GPx, GR and Cat calculation was 7, while BFGS 484 was the training algorithm, and the activation functions for hidden and output layers were logistic. Experimental results were well predicted by the ANN model for a number of the process variables. The ‘goodness of fit’ of the ANN model is shown in Table 2. Namely, *r*^2^ values were close to 1 (0.976; 0.957; 0.923; 0.960 and 0.967 for SR, T–SH, GPx, GR and Cat, respectively), which showed a good prediction capability of the model. Additionally, *χ*^2^ and RMS of SR and T–SH are augmented due to high nominal values obtained by these antioxidant assays, but *χ*^2^, MBE, RMSE and MPE values of other assays were close to 0, which indicates a good model fit.

The obtained overall skewness parameter was close to zero (this parameter measures the deviation of the distribution from normal symmetry), which means that the distribution of residual values is close to symmetrical. Kurtosis measures the “peakedness” of a distribution, and the results of the kurtosis test show a good approximation to the normal distribution. The kurtosis of SR indicated a peaked distribution of residuals, which was also observed by *χ*^2^ and RMSE values. The residual analysis showed that the mean of residuals were close to zero, and the standard deviation was between 0.159 and 31.970. The mean and SD values of SR and T–SH are also increased due to high variance, as indicated by the results displayed in Table 2. These results showed a good approximation to a normal distribution around zero with a probability of 95% (2 × SD), which means that the developed ANN model had a good generalization ability to predict the observed experimental antioxidant data.

### 2.4. Sensitivity Analysis

**Figure 6:**
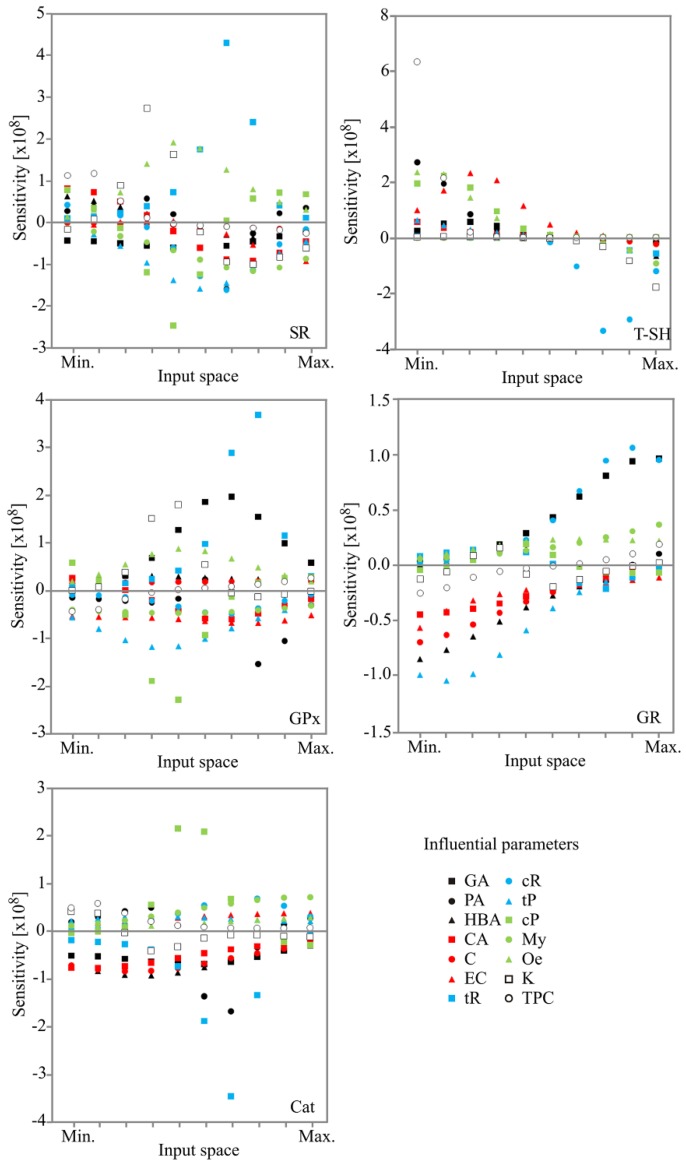
Sensitivity analysis—the influence of the input over the output variables. TPC—Total phenolic content; GA—Gallic acid; PA—Protocatechuic acid; HBA—4-Hydroxy benzoic acid; CA—Caffeic acid; C—Catechin; EC—Epicatechin; tR—*trans*-Resveratrol; cR—*cis*-Resveratrol; tP—*trans*-Piceid; cP—*cis*-Piceid; My—Myricetin, Qe—Quercetin; K—Kaempferol.

The influence of the inputs over the output variables, i.e., calculated changes of output variables for infinitesimal changes in input variables, is shown on Figure 6. The obtained values corresponded to the level of experimental errors, and also showed the influence of phenols content on AA of wines expressed by SR, T–SH, GPx, GR and Cat.

Though sensitivity analysis is used to show the influence of the inputs, it also points out the importance of an input variable at a given point in the input space [33]. While small changes in tR, Qe and K contents predominantly affected SR in a positive manner at the centre of the input space, small changes in cP, cR and tP contents actually displayed an opposite effect (Figure 6). At the minimum of the input space T–SH has been positively affected by small changes in TPC. However, at the centre of an input space, infinitesimal changes in cR content were linked with a negative impact. GPx was mostly positively affected by the infinitesimal changes in tR close to the upper end of input space. In the same way tR affected SR (Figure 6). In other words, small changes in tR content had the highest positive effect on GPx and, consequently, SR. Positive effect of tR on GPx activity has been previously claimed. For example, Sadi et al. [34], reported that hepatic GPx activity was increased in diabetic rats upon treatment with resveratrol, while Ourique et al. [35] observed the same effect of resveratrol in the testes of T3–induced hyperthyroid rats. In the centre of input space GA exhibited the same pattern of positive influence on GPx activity and SR, while cP content negatively affected both parameters. These findings actually support an observation that GPx is mainly responsible for SR increase in H_2_O_2_ stressed yeast cells pre–treated with the examined wine samples. Furthermore, close to the upper end of the input space, GR was mostly positively affected by cR and GA contents. On the contrary, at the minimum of the input space, the same parameter (GR) was negatively affected by tP and HBA. While small changes in cP content mostly positively affected Cat, infinitesimal changes in tR and PA contents were responsible for negative effects, at the centre of the input space. Such a finding is in accordance both with PCA and experimentally performed measurements.

## 3. Materials and Methods

### 3.1. Chemicals and Reagents

Folin–Ciocalteu’s phenol reagent, DPPH, 5,5′-dithiobis(2-nitrobenzoic acid) and reduced L-glutathione were purchased from Sigma-Aldrich (Steinheim, Germany). A Ransel kit was obtained from Randox Laboratories (Crumlin, UK). The BIOXYTECH kit was obtained from OXIS International (Foster City, CA, USA). Finally, *Saccharomyces cerevisiae* (Zymaflore RX60 Vin Rouge) was purchased from Laffort-BP 17 (Bordeaux, France).

### 3.2. Wine Samples

The commercial Merlot wine, along with red wine samples obtained from recognized clones (VCR 1 and VCR 101) of the same variety (vintage 2011), were provided by developing sector of “Plantaže 13. juli” A.D. winery (Podgorica, Montenegro). The samples were labeled as Comm, C I and C II, for the samples of the commercial wine, VCR 1 and VCR 101 clone wines, respectively. All the analyzed samples were produced under the same conditions applying standard vinification procedure [36]. Prior to analysis, they were stored at 10 °C in the dark and analyzed immediately after opening.

### 3.3. Total Phenolic Content

TPC was determined by slightly modified Folin–Ciocalteu’s assay [37]. The absorbance of wine samples (six-fold diluted with water) was recorded at 740 nm using a UviLine 9400 UV–VIS spectrophotometer (SI Analytics, Mainz, Germany). TPC was calculated using a calibration curve with gallic acid (125–1000 μg/mL). All samples were analyzed in triplicate and processed simultaneously with calibration curve. The obtained results are expressed as mg gallic acid equivalents per L (mg GAE/L).

### 3.4. Total Flavonoid Content

TFC was determined according to the method of Chia-Chi et al. using aluminum chloride as a reagent [38]. The absorbance was measured at 410 nm with a Cintra40 UV–VIS spectrophotometer (GBC, Hampshire, IL, USA). All samples were analyzed in triplicate. Calibration curve, using rutin as standard (50–500 μg/mL), and samples were processed simultaneously. The results are expressed as mg rutin equivalents per L.

### 3.5. Total Monomeric Anthocyanin Content

TMA content was determined by pH differential method according to Lee et al. [39]. The absorbance was measured on a GBC Cintra 40 UV–VIS spectrophotometer at 520 and 700 nm. All samples were analyzed in triplicate. Calibration curve, using cyanidin-3-glucoside as standard (50–500 μg/mL), and samples were processed simultaneously. The results are expressed as mg cyanidin-3-glucoside (Cyd-3-glu) equivalents per L.

### 3.6. Anti–DPPH Radical Activity

Anti–DPPH radical activity of the wine samples was measured according to the previously described method [38]. Five different dilutions of each analyzed sample were made. Each dilution was made in triplicate. The absorbance was recorded at 517 nm on a SI Analytics UviLine 9400 UV–VIS spectrophotometer. The obtained results are expressed as IC_50_^−1^ values, representing the reciprocal volume of the wine sample able to scavenge 50% of DPPH radical.

### 3.7. Cellular Assay for the Evaluation of Antioxidant Activity

This assay was used to evaluate AA of analyzed wines against oxidative stress caused by H_2_O_2_ in *Saccharomyces cerevisiae*. Yeast cells, grown in liquid Sabouraud dextrose broth, were transferred to a fresh broth at the exponential phase. The broth was diluted with fresh medium to get 0.2 of absorbance (A_600_). The optimal concentration of the wine sample was determined using the serially–diluted wine samples in the concentration range 2–8 mL/100 mL. The lowest concentration which improved cell growth (compared to the yeast cells exposed to 0.75 mM of H_2_O_2_) was selected for further study. Three control groups were used: ethanol control (Cont, cells treated with 13% ethanol), wine control (Comm, C I, C II, i.e., the cells treated only with respective wine sample) and H_2_O_2_ control (Cont + H_2_O_2_ cells treated with H_2_O_2_). Each mixture contained wine at the final concentration of 4 mL/100 mL; H_2_O_2_ was then adjusted to 0.75 mM 15 min after the addition of wine. All samples and controls were incubated in a thermostable shaker (IKA KS 4000 Thermostabile shaker, Thermo Scientific, Waltham, MA, USA) for 1 h at 28 °C with constant shaking at 160 rpm. After serial ten-fold dilution, cell viability was determined by plating on Sabouraud dextrose agar. The number of colonies on each plate was about one hundred. Afterwards, Petri dishes were incubated at 28 °C for 72 h. Subsequently, the number of colonies was counted. One hundred percent survival rate (SR) is attributed to the number of colonies noted on the ethanol control plate.

### 3.8. Preparation of Cell–Free Extracts

For determination of total sulfhydryl (T–SH) groups content and enzymatic assays, the yeast cells were prepared according to Lingua et al. [32]. In brief, yeast cells were lysed in lysis buffer (50 mM Tris–HCl, 150 mM NaCl, 50 mM EDTA, pH 7.2) using 2 vol of 425–600 microns glass beads for cells breaking. Cells were alternately vortexed and cooled on ice for 1 min, 8 times. After cell debris was removed by centrifugation (14,000× *g* for 15 min at 4 °C), the supernatant used for further analyses was obtained. The protein concentrations were determined using BCA™ Protein Assay Kit, Thermo Scientific (Waltham, MA, USA).

### 3.9. Determination of T–SH Groups

The content of T–SH groups was measured using Ellman’s reagent (DTNB) [39]. The samples/standard (20 μL) were mixed with 75 μL of dilution buffer (30 mM Tris–HCl, 3 mM EDTA, pH 8.2), 25 μL of 3 mM DTNB reagent and 400 μL of methanol. After centrifugation at 3000× *g* for 5 min at room temperature, the supernatants were transferred into a flat-bottom microplate. Absorbance was measured at 412 nm with a WALLAC 1420—Victor^2^ Multilabel Counter, LKB (London, UK). Finally, quantification was done according to the following standard curve (0–1000 μM GSH standard solutions). All samples were analyzed in triplicate.

### 3.10. Determination of GPx, GR and Cat Activity

GPx and GR activities were measured using a Ransel kit (Randox Laboratories) and a BIOXYTECH kit (OXIS International) respectively, according to the manufacturer’s instructions. The millimolar extinction coefficient of nicotinamide adenine dinucleotide phosphate (NADPH) at 340 nm (6.22 mM^−1^ cm^−1^) was used for calculations of GPx and GR activities which were expressed as mU per mg protein. Determination of Cat activity was based on monitoring of H_2_O_2_ decomposition, whereby the decrease in absorbance at 240 nm was proportional to Cat activity [40]. The millimolar extinction coefficient of H_2_O_2_ at 240 nm (43.6 × 10^−3^ mM^−1^ cm^−1^) was used for calculations of Cat activity expressed as U per mg protein. All assays were done in triplicate.

### 3.11. Statistical Analyses

The data were processed statistically using the software package STATISTICA 10.0 (StatSoft Inc., Tulsa, OK, USA). All analyses were done in three replicates. The obtained results are expressed as the mean value with standard deviation (SD). All observed samples were checked for variance equality (using Levene’s test) and normal distribution (using Shapiro–Wilk’s test). Analysis of variance (ANOVA) with Tukey’s HSD post hoc test for comparison of the sample means were used to explore the variations of TPCs and parameters related to AA of three wine samples.

### 3.12. PCA Data Analysis

PCA is performed by eigenvalue decomposition of a data correlation matrix [41]. This transformation is defined in such a way that the first component has the largest variance. PCA is used to achieve maximum separation among clusters of parameters, evidencing spatial relationship between processing parameters, and enabled a differentiation between the samples [42].

### 3.13. ANN Modeling

ANN model was used to anticipate the exploratory values for SR, T–SH, GPx, GR and Cat. To improve the behavior of the ANN, both input and output data were normalized. A multi–layer perceptron model (MLP) that consisted of three layers (input, hidden and output) has been proven quite capable of approximating nonlinear functions [43], giving the reason for choosing it within this work. The Broyden–Fletcher–Goldfarb–Shanno (BFGS) algorithm, utilized for ANN modeling, was used as the training algorithm.

### 3.14. Sensitivity Analysis

Sensitivity analysis is a sophisticated technique necessary for studying of the effects of observed input variables along with uncertainties in obtained models and general network behavior. ANN was tested by sensitivity analysis, aiming to determine whether and under which circumstances the obtained model might result in a well adapted system [44].

### 3.15. Accuracy of the Models

The numerical verification of the developed models was tested using coefficient of determination (*r*^2^), reduced chi-square (*χ*^2^), mean bias error (MBE), root mean square error (RMSE) and mean percentage error (MPE). These commonly used parameters were calculated as follows:

(1) $$\chi^{2}=\frac{\sum_{i=1}^{N}{\left(x_{\exp,i}-x_{pre,i}\right)}^{2}}{N-n},\;\mathrm{RMSE}={\left[\frac{1}{N}\cdot \sum_{i=1}^{N}{\left(x_{pre,i}-x_{\exp,i}\right)}^{2}\right]}^{1/2}$$

(2) $$\mathrm{MBE}=\frac{1}{N}\cdot \sum_{i=1}^{N}\left(x_{pre,i}-x_{\exp,i}\right),\;\mathrm{MPE}=\frac{100}{N}\cdot \sum_{i=1}^{N}\left(\frac{\left|x_{pre,i}-x_{\exp,i}\right|}{x_{\exp,i}}\right)$$

where *x*_exp,*i*_ stand for the experimental values while *x_pre,i_* represents the predicted values calculated from the model for these measurements. Finally, *N* and *n* are the number of observations and constants, respectively.

## 4. Conclusions

This study demonstrated the antioxidant activity of three Merlot wines. Their protective effects corresponded to the abundance of phenolic components. Specifically, H_2_O_2_–stressed yeast cells pre–treated with C I wine that had the highest contents of gallic acid, catechin, resveratrol as well as its glucosides, showed the best survival rate. Additionally, the obtained results suggested that protective effects of wine samples were primarily due to glutathione peroxidase activity. Finally, using an artificial neural network we developed a rapid and accurate method for prediction of antioxidant activity according to phenolic content in wine with high prediction accuracy (*r*^2^ = 0.978). Sensitivity analysis of the developed artificial neural network model provides an insight into the complexity of the impact that variations in the concentrations of specific phenolic components have on the antioxidant defense system.

By combining examination of cellular effects of red wines with advanced mathematical modeling, this study brings a new approach to research not only of wines, but also of other natural products that are classified into the functional food. It also presents a strong starting point in the analysis of the effects that natural products may have on molecular level in live cells. This will be of special interest for the future studies in the field of functional foods.

## Figures and Tables

**Table 1:** The total phenolic, flavonoid and monomeric anthocyanin content determined in examined wines.

Content		Comm	C I	C II
Total phenolic content	TPC	2988.6 ± 142.2 ^a^	3255.7 ± 90.8 ^a,b^	3337.9 ± 115.4 ^b^
Total flavonoid content	TFC	376.0 ± 11.2 ^a^	393.2 ± 5.8 ^a^	397.2 ± 9.2 ^a^
Total monomeric anthocyanin content	TMA	215.2 ± 4.1 ^a^	203.6 ± 4.5 ^a^	230.3 ± 6.6 ^b^

All values are expressed as mean ± SD. Value for total phenolic, total flavonoid and total monomeric anthocyanin content are expressed as gallic acid, rutin and cyanidin-3-glucoside equivalents (mg/L). Values followed by different letters (a and b) in the same row are significantly different (*p* < 0.05), according to Tukey’s HSD test. Comm—commercial wine; C I—clone I wine; C II—clone II wine.

**Table 2:** The “goodness of fit” tests and the residual analysis for the optimal ANN.

	*χ* ^2^	RMSE	MBE	MPE	*r* ^2^	Skew.	Kurt.	Mean	SD	Var.
SR	29.678	5.136	0.713	4.230	0.976	−0.565	2.797	0.713	5.183	26.867
T–SH	1115.430	31.488	−2.696	0.950	0.957	−0.607	0.630	−2.696	31.970	1022.080
GPx	0.027	0.156	0.005	1.013	0.923	0.688	−0.183	0.005	0.159	0.025
GR	2.935	1.615	−0.103	1.116	0.960	0.034	−1.049	−0.103	1.643	2.698
Cat	6.120	2.332	−0.004	2.426	0.967	0.642	0.635	−0.004	2.377	5.649

*χ*^2^—reduced chi-square; RMSE—root mean square error; MBE—mean bias error; MPE—mean percentage error; *r*^2^—coefficient of determination; Skew.—skewness; Kurt.—kurtosis; Mean—average of the residual; SD—standard deviation; Var.—variance; SR—survival rate; T–SH—total sulfhydryl groups; GPx—glutathione peroxidase; GR—glutathione reductase; Cat—catalase.
